# High-resolution imagery acquired from an unmanned platform to estimate biophysical and geometrical parameters of olive trees under different irrigation regimes

**DOI:** 10.1371/journal.pone.0210804

**Published:** 2019-01-22

**Authors:** Giovanni Caruso, Pablo J. Zarco-Tejada, Victoria González-Dugo, Marco Moriondo, Letizia Tozzini, Giacomo Palai, Giovanni Rallo, Alberto Hornero, Jacopo Primicerio, Riccardo Gucci

**Affiliations:** 1 Dipartimento di Scienze Agrarie, Alimentari e Agro-ambientali, Università di Pisa, Pisa, Italy; 2 Instituto de Agricultura Sostenible (IAS), Consejo Superior de Investigaciones Científicas (CSIC), Córdoba, Spain; 3 School of Agriculture and Food, Faculty of Veterinary and Agricultural Sciences (FVAS), University of Melbourne, Melbourne, Australia; 4 Department of Infrastructure Engineering, Melbourne School of Engineering (MSE), University of Melbourne, Melbourne, Australia; 5 CNR-IBIMET–Consiglio Nazionale delle Ricerche, Istituto di Biometeorologia, Firenze, Italy; 6 Department of Geography, Swansea University, Swansea, United Kingdom; Georgia Southern University, UNITED STATES

## Abstract

The experiments were conducted in a fully-productive olive orchard (cv. Frantoio) at the experimental farm of University of Pisa at Venturina (Italy) in 2015 to assess the ability of an unmanned aerial vehicle (UAV) equipped with RGB-NIR cameras to estimate leaf area index (LAI), tree height, canopy diameter and canopy volume of olive trees that were either irrigated or rainfed. Irrigated trees received water 4–5 days a week (1348 m^3^ ha^-1^), whereas the rainfed ones received a single irrigation of 19 m^3^ ha^-1^ to relieve the extreme stress. The flight altitude was 70 m above ground level (AGL), except for one flight (50 m AGL). The Normalized Difference Vegetation Index (NDVI) was calculated by means of the map algebra technique. Canopy volume, canopy height and diameter were obtained from the digital surface model (DSM) obtained through automatic aerial triangulation, bundle block adjustment and camera calibration methods. The NDVI estimated on the day of the year (DOY) 130 was linearly correlated with both LAI and leaf chlorophyll measured on the same date (R^2^ = 0.78 and 0.80, respectively). The correlation between the on ground measured canopy volumes and the ones by the UAV-RGB camera techniques yielded an R^2^ of 0.71–0.86. The monthly canopy volume increment estimated from UAV surveys between (DOY) 130 and 244 was highly correlated with the daily water stress integral of rainfed trees (R^2^ = 0.99). The effect of water stress on the seasonal pattern of canopy growth was detected by these techniques in correspondence of the maximum level of stress experienced by the rainfed trees. The highest level of accuracy (RMSE = 0.16 m) in canopy height estimation was obtained when the flight altitude was 50 m AGL, yielding an R^2^ value of 0.87 and an almost 1:1 ratio of measured *versus* estimated canopy height.

## Introduction

Although olive (*Olea europaea* L.) is a drought tolerant species, irrigation is widely used in modern, high density olive orchards due to its beneficial effects on growth, yield components and oil quality [1, 2, 3, 4]. Shoot growth, leaf area and canopy volume are very sensitive to water stress as they affect tree water requirements [5, 6, 7, 8]. Pérez-López et al. [9] compared the effects of different irrigation regimes on shoot growth and canopy volume of young olive trees (3-year-old at the beginning of the trial) and found significant differences only in the third year of the experiment. They attributed the lack of significant differences in canopy volume in the first two years to the low accuracy of the measurement method and to the high variability within each treatment; as canopy volume progressively increased differences became evident. Another study conducted in a high-density olive orchard showed marked effects of water deficit on canopy volume and leaf area index (LAI), with the highest values measured in fully-irrigated trees and the lowest ones in trees subjected to regulated deficit irrigation [10]. They concluded that the increase in canopy volume could be used as an indicator to quantify the effect of water stress on vegetative growth. In that work differences in vegetative parameters between irrigation treatments were detected also thanks to the precise and accurate methods used for estimating both LAI and tree canopy volume [11, 12]. The reduction in vegetative growth induced by water deficit can be used effectively to control canopy size in mature trees, especially in high and very high density orchards [6]. Moreover, both LAI and tree canopy volume are directly related to solar radiation interception, water consumption and potential productivity of olive trees [13, 14] and provide valuable information for both pest and pruning management [15, 16].

LAI can be calculated by either direct measurements, involving destructive sampling, or indirect ones, based on hemispherical photography or radiative transfer models [17, 18]. Indirect methods are currently preferred as they are less expensive and time consuming than destructive ones. The tree canopy can be characterized by manual and electronic methodologies. The most widely used manual method for canopy volume estimation of individual trees is the ellipsoid method [11, 19], whereby the volume is obtained by measuring crown diameters and canopy height, assuming an ellipsoidal shape of the tree canopy. Alternatively, the projected area of the tree crown can be used to estimate canopy volume [10]. However, manual measurements are time consuming also due to the irregular shape of the trees. For instance, the most widespread training system in high density olive orchard is the vase, characterized by an empty volume in the central part of the canopy, which improves both light penetration and air circulation [20]. Current advances in sensor technologies offer a number of alternatives to the traditional manual-based measurements [21]. Electronic measurement methods use sensors that can measure geometrical parameters, such as the canopy volume or density. The tree canopy can be described using ultrasonic sensors, optical sensors or LiDAR (light detection and ranging) based sensors [21, 22]. LiDAR systems are very precise and they are able to create three-dimensional models of the orchard and characterize the olive tree canopy architecture [23, 16]. However, these systems are still uncommon in commercial orchards due to their cost, especially when large and rugged areas are to be scanned.

Several remote sensing techniques are currently being used for estimating LAI and canopy volume as an alternative to the on-ground field measurements. In the last decade unmanned aerial vehicles (UAV) have been proposed for agricultural applications due to their great flexibility in flight scheduling, low operational costs and the increasing availability of dedicated, low-cost, miniaturized sensors. Previous studies reported that vegetative spectral indices derived from multispectral cameras mounted on UAVs were well related to direct measurements of LAI in olive [24, 25]. UAV-based remote sensing platforms have been also used in olive orchards for estimating plant architecture through the computation of a digital surface model (DSM) for both isolated trees and hedgerows [26, 27]. Briefly, a DSM is a digital representation of a topographic surface accounting for the height of the upper surface of the terrain and of the objects thereon, which can be used to obtain tree height information. A digital terrain model (DTM) represents the topographic surface including only the height of terrain surface, thus excluding the height of the objects on it. Zarco-Teiada et al. [28] estimated the tree height in an olive orchard using a low-cost RGB camera, modified for color-infrared detection, mounted on a UAV platform. They also evaluated the effects of the spatial resolution of the input images on the digital surface model (DSM) obtained by photo-reconstruction. Authors obtained root mean square error (RMSE) values of 0.35 m when estimating the tree heights from imagery of 0.05 m spatial resolution used as input for the DSM generation.

The overall objective of this study was to assess the ability of UAVs equipped with RGB-NIR cameras to estimate leaf area index, tree height, tree canopy diameter and canopy volume of olive trees which were either irrigated or rainfed. In particular, we propose a methodology for the tree canopy volume estimation based on the processing of DTM and DSM raster files obtained from a low-cost RGB camera.

## Materials and methods

### Plant material and site characteristics

**Fig 1:**
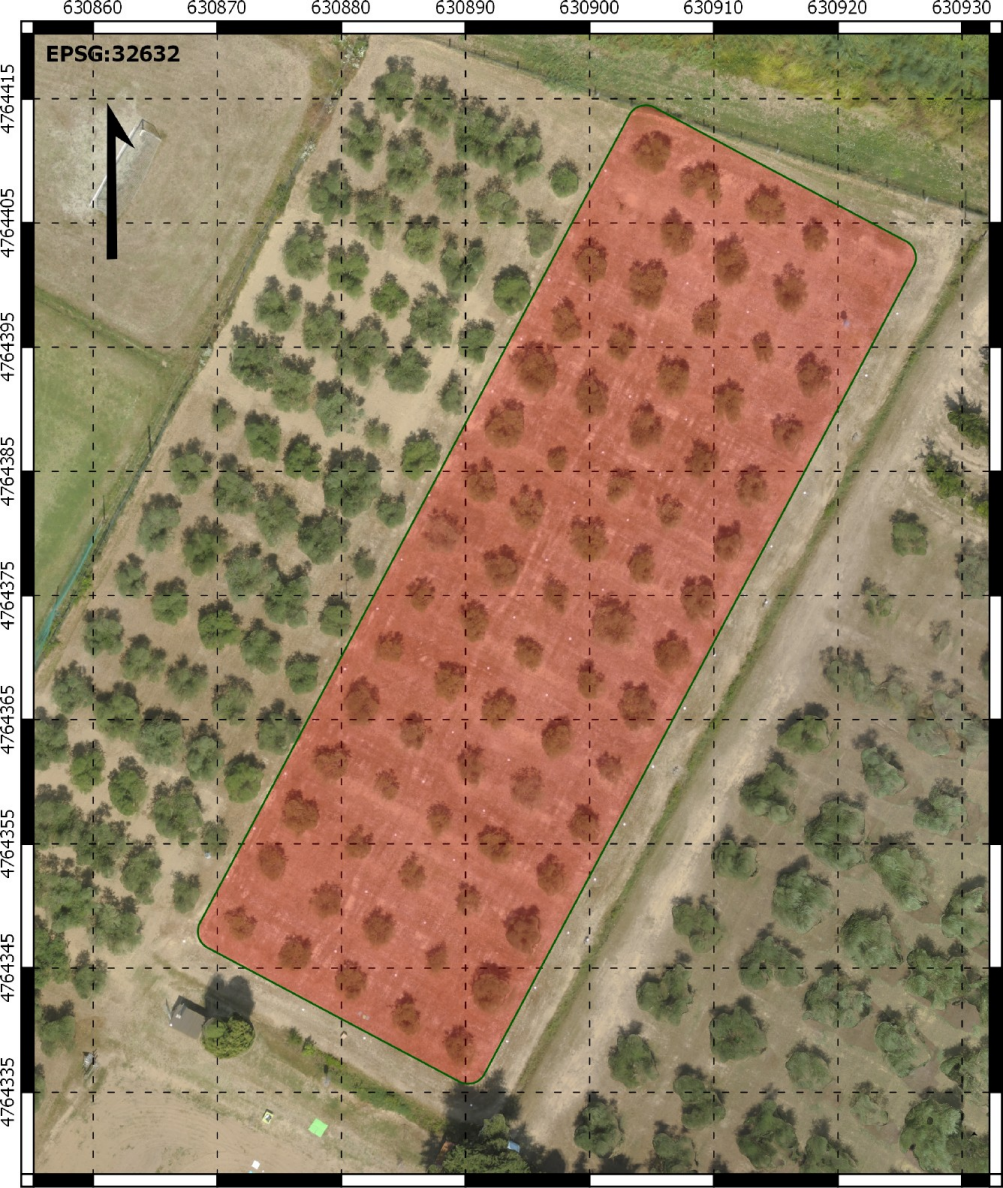
Aerial view of the olive orchard (cv. Frantoio) monitored by an UAV equipped with RGB-NIR cameras.

Experiments were conducted in a mature, irrigated olive orchard of cv. Frantoio at the experimental farm of University of Pisa at Venturina (Italy) in 2015. Olive trees were planted at a spacing of 5 x 5 m in April 2009 (Fig 1). The soil was a deep (1.5 m), sandy-loam (ISSS classification), consisting of 60% sand, 15% clay, and 25% silt. The climate at the study site was sub-humid Mediterranean [1], with an annual mean temperature and annual rainfall of 15.7°C and 676 mm, respectively (means of 28 years, 1990–2017). Climatic conditions over the study period were monitored using an iMETOS IMT 300 weather station (Pessl Instruments GmbH, Weiz, Austria). Reference annual evapotranspiration (ET_0_), calculated according to the Penman-Monteith equation, was 952 mm. The annual and summer (21 June–22 September) precipitations were 809 and 39 mm, respectively. Cultural practices and monitoring of phenological parameters, including the estimation of full bloom date, were performed as previously reported [1]. In 2015 full bloom, manually estimated when 70% of inflorescences of selected branchlets showed at least 50% of flowers open, occurred on DOY 146. The olive orchard was divided into five plots, each included 15 trees arranged in three rows of five trees. Three plots were assigned to the full irrigation treatment, the remaining two to the rainfed treatment. Only the inner trees of the central row were used for monitoring tree water status.

### Irrigation scheduling and tree water status

Trees were either rainfed (RF) or irrigated (IR) using a subsurface drip irrigation system (1.6 L h^-1^ pressure-compensated drippers spaced at 0.6 m). The irrigation period lasted from DOY 182 through DOY 273, during which IR trees received water 4–5 days a week (5 to 7 h per day) for a total volume of 1348 m^3^ ha^-1^, corresponding to 3370 L per tree. The RF condition was partially relieved by one complementary irrigation of 19 m^3^ ha^-1^ on DOY 273. That was necessary to prevent tree dehydration and permanent damage. Irrigation volumes were calculated on the basis of the effective evapotranspiration using a crop coefficient (K_c_) of 0.55 and a coefficient of ground cover (K_r_) of 0.46 [1, 29]. Tree water status was determined by measuring stem water potential (SWP) during the dry season at 7–10 d intervals. SWP was measured after blocking transpiration of leaves inserted near the main scaffolds of the tree [30]. The leaf was enclosed for at least 45 min in a non-transpiring shaded bag to block transpiration and then sampled to determine SWP once the potential reached equilibrium with the xylem. Leaves were excised with a razor blade, immediately put in the chamber cylinder (Tecnogas, Pisa, Italy), which was then pressurized with nitrogen gas at a maximum rate of 0.02 MPa s^−1^ [1]. In order to consider the fluctuations in tree water status during the irrigation period, the water stress integral (WSI) was calculated from the SWP data, as reported in Myers [31]. Fertigation was used to supply mineral nutrients in spring before irrigation treatments were started. Each tree received a total of approximately 80 g of each N, P_2_O_5_, and K_2_O.

### Leaf area index, leaf chlorophyll content, canopy volume and trunk cross sectional area

Leaf area index (LAI) measurements were derived by means of a LAI-2000 optoelectronic sensor (LI-COR, Lincoln, Nebraska, USA) on DOY 130 in 2015. Due to the heterogeneous structure of the canopies within the orchard, a stand-alone protocol was followed [18]. The sensor used a 90° view cap to block the operator from the sensor’s view and was held at a distance of at least 0.4 m above the ground, in order to leave approximately 0.3 m from the base of the canopy.

A sample of 30 leaves with variable greenness index was selected on DOY 130 in 2015 to determine the correlation between SPAD readings (Konica Minolta, Inc., Osaka, Japan) and the actual chlorophyll concentration determinations. Chlorophyll was extracted using N,N-dimethylformamide according to the method described by Moran and Porath [32], and total chlorophyll (a+b, Cab) concentration (μg cm^-2^ of leaf area) calculated based on the equations provided by Lichtenthaler and Wellburn [33]. Trichomes were stripped off by applying adhesive tape on the abaxial side of the leaf before extraction. The SPAD readings confirmed to be a reliable proxy of chlorophyll concentration per unit of leaf area (Cab = 0.8271 SPAD– 12.8; R^2^ = 0.84) (S1 Fig). This equation was then used to convert the SPAD data into leaf chlorophyll concentrations. The readings were taken on DOY 130 in 2015 on 30 fully-expanded leaves for each of the 23 monitored trees.

Tree canopy volume (m^3^) was calculated on DOY 130 and DOY 188 in 2015, and DOY 63 in 2016, assuming an ellipsoidal shape:

$$Volume\;of\;ellipsoid=\frac{4\pi }{3}\;a\;b\;c$$ (1)

where a, b and c are the three semi-axes of the ellipsoid. When applied to the olive tree the above equation became:

$$Canopy\;volume=\frac{4\pi }{3}\;\frac{D1}{2}\;\frac{D2}{2}\;\frac{Ht-Hb}{2}$$ (2)

where D1 and D2 are crown diameters, Ht is tree height, Hb is the lowest canopy height from the ground. The trunk cross sectional area (TCSA) was calculated from the circumference of the trunk at 0.40 m from ground, measured on DOY 39 and 309 in 2015.

### Multispectral imagery acquisitions from the unmanned platform

The acquisition campaign was performed using a S1000 UAV octocopter (DJI, Shenzhen, China) able to fly autonomously over a predetermined waypoint course. The S1000 was equipped with a 2-axis stabilized gimbal equipped with a consumer photo-camera (RGB) and a multispectral camera (NIR-RG). The RGB camera was a Coolpix P7700 (Nikon, Shinjuku, Japan) embodying a 12.2-megapixel CMOS sensor, whereas the NIR-RG multispectral camera was a Tetracam ADC-lite (Tetracam, Inc., Gainesville, FL, USA) equipped with a 3.2-megapixel CMOS sensor. Images were recorded in the visible red (R), green (G) and near-infrared (NIR) domain with nominal wavelengths of 520–600, 630–690, and 760–900 nm, respectively. Six flights were made on DOY 283 (2014), DOY 130, 188, 244 and 343 (2015) and 63 (2016). Images were acquired at noon under clear sky conditions, the flight altitude was 70 m above ground level (AGL), except for the flight performed on DOY 63 in 2016 (50 m AGL), flying in all cases at 3.5 m s^-1^ speed (Table 1). On DOY 188 (2015) and 63 (2016), before the UAV flight, a set of 67 and 14 ground control points (GCPs), respectively, were placed in the orchard and georeferenced using a Leica GS09 real time kinematic GPS (Leica Geosystems A.G., Heerbrugg, Switzerland) able to achieve a 3D resolution of 0.02 m (Table 1). For the other four flights no GCP measurements were made in the field with a GNSS-RTK, but Manual Tie Points (MTP) were added instead; this was performed by defining a marker on relevant images and entering its latitude, longitude and altitude values manually.

**Table 1:** Image acquisition.

DOY	Year	Flight altitude (m)	GCPs	Images acquired	Images used	GSD (cm)	Images overlap
130	2015	70	6*	88	88	2.14	5+
188	2015	70	67	150	150	2.20	5+
63	2016	50	14	59	59	1.44	5+

Flight altitude, number of ground control points (GCPs), number of RGB images acquired and used for the DSM generation, ground sample distance (GSD) and image overlap at each date of flight. Images overlap indicates the number of overlapping images computed for each pixel of the orthomosaic.

* Manual Tie Points (More details are reported in the Material and Methods section).

### Image processing methods

**Fig 2:**
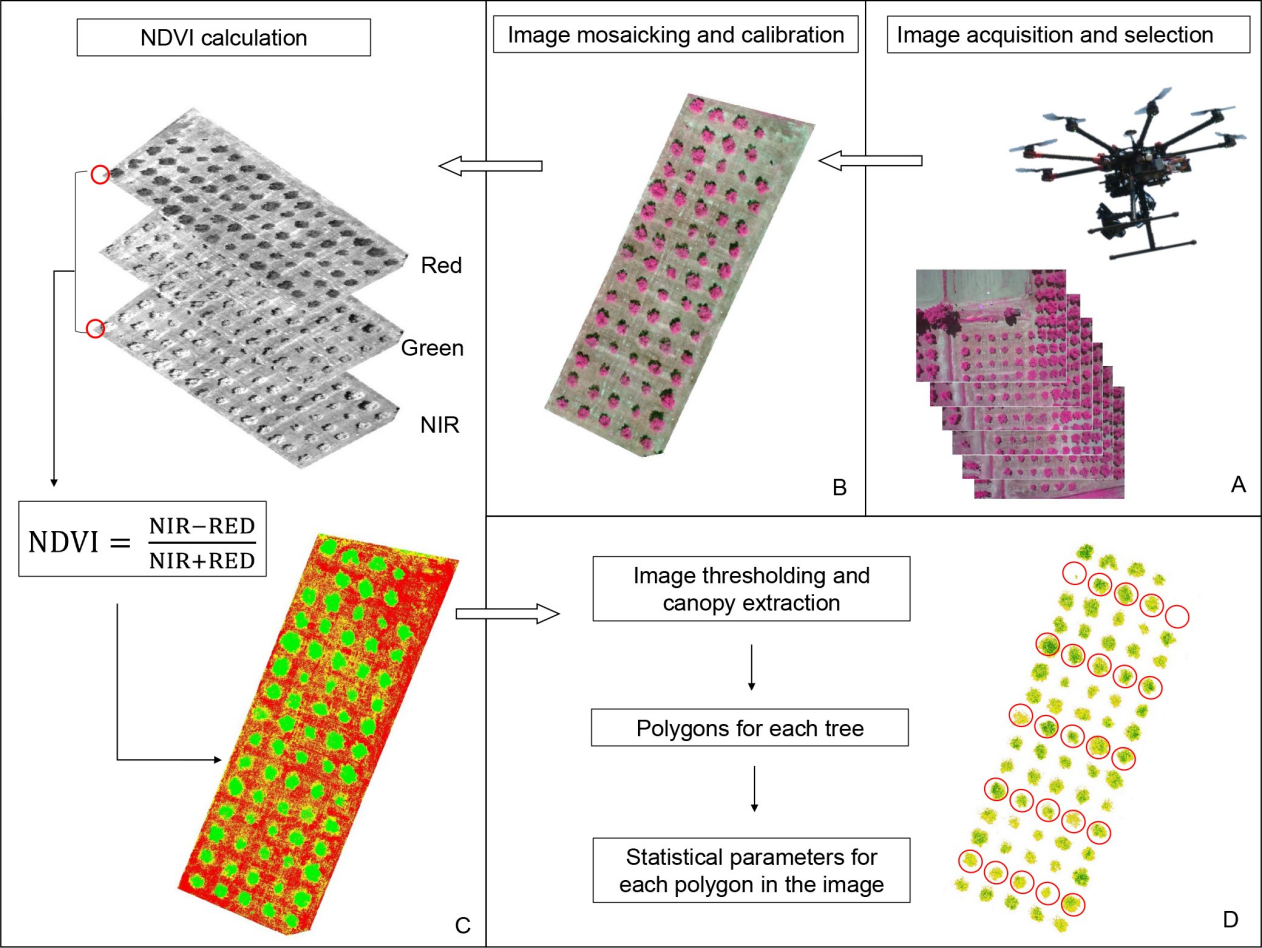
Flowchart of the procedure for the NDVI calculation using multispectral images acquired by an UAV.

The multispectral images were first mosaicked using Autopano Giga 3.5 Software (Kolor SARL, Challes-les-Eaux, France), then georeferenced and orthorectified using the ground referenced points (ArcGIS software, ESRI, Redlands, CA, USA) (Fig 2). The NDVI [34] was calculated by means of the map algebra technique implemented in ArcGIS software (ESRI, Redlands, Ca, USA) using the following equation:

$$NDVI=\frac{NIR-RED}{NIR+RED}$$

where NIR and RED are the values of the reflectance in the near-infrared (630–690 nm) and red (760–900 nm) bands, respectively.

**Fig 3:**
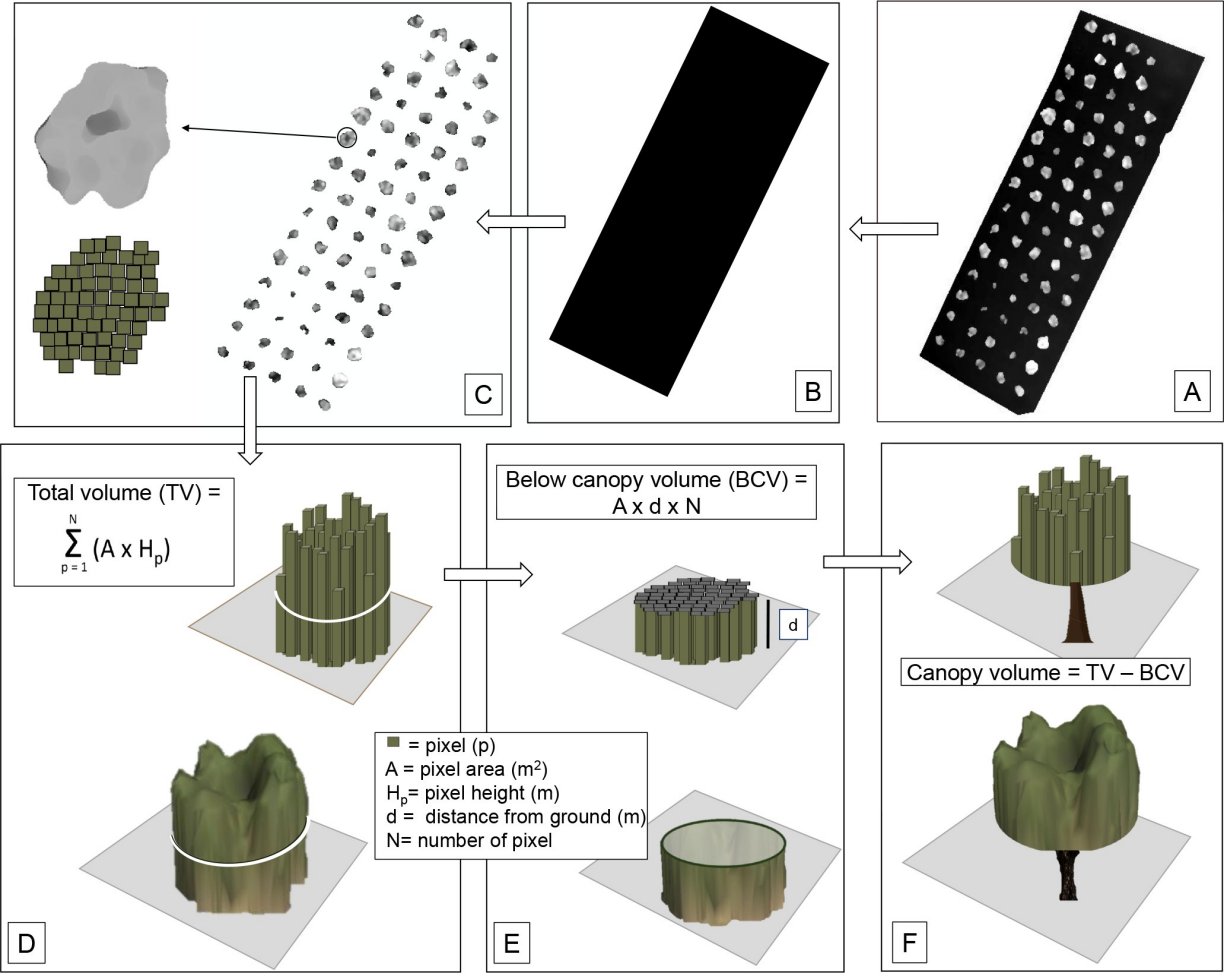
Schematic workflow used to estimate the tree canopy volume. (A) DSM generated through the SfM technique. (B) DTM obtained from DSM in GIS ambient by triangulation of the points located in the inter-rows and intra-rows zones. (C) nDSM obtained by the difference A minus B, and containing only tree canopies. (D) total tree volume including the below canopy volume; E) below canopy volume calculation. (F) canopy volume obtained by subtracting the below canopy volume to the total volume. More details are reported in the Materials and Methods section.

The three-dimensional tree canopy volume was reconstructed starting from the digital surface model (DSM) obtained through automatic aerial triangulation, bundle block adjustment and camera calibration methods using Pix4D version 3.1.23 (Lausanne, Switzerland) as in Zarco-Tejada et al. [28]. GCPs were added to improve the accuracy, ensuring absolute retrievals for the DSM generated, maintaining consistency between the different flights performed. In detail, the DSM generated from the point cloud (Fig 3A) was then processed in ArcGIS to obtain a digital terrain model (DTM) of the orchard (Fig 3B) to retrieve the height of each three-dimensional axes of the canopy point above the ground. The normalized DSM (nDSM), consisting in a raster including only the pixels of the tree canopy (Fig 3C) was obtained by subtracting the DTM to DSM using the raster calculator tool of the ArcGis software. The volume for each pixel (from the ground to the maximum height of the canopy) was obtained by multiplying their area by the corresponding height value. The total volume of each tree (from the ground to the maximum height of the canopy) was obtained by adding all the canopy pixels (Fig 3D). At this point, the net canopy volume (Fig 3F) was calculated by subtracting to the total volume of each tree the volume comprised between the ground and the lowest part of the canopy (Fig 3E). This latter volume was calculated for each tree by using two distances (d) of the canopy from the ground manually measured using a graduate rod: a) an average value of 0.7 m (mean of all trees of the orchard); b) the actual measured value for each tree.

Canopy height and diameter for each tree were extracted using an automated object-based method. The very high-resolution images acquired allowed the identification and delimitation of each tree crown independently; object-based image segmentation methods were based on both Niblack's thresholding method [35] and Sauvola and Pietikäinen's binarization techniques [36] to separate tree crowns from the background. Image segmentation was automatically conducted splitting the DSM into multiple regions, according to the height, that made possible to identify single tree crowns without using any watershed analysis, given no overlapping crowns were recognised. The height was calculated using the average height of the terrain surrounding each tree and the maximum height of each tree, obtaining the absolute height as the difference of both. The diameter was calculated assuming the circularity of the tree surface seen from above; therefore, the projected area of each tree was obtained using a height threshold which delimited soil and tree components.

### Statistical analysis

In order to test the ability of UAV imagery to estimate the biophysical and geometrical tree parameters, the data sets obtained from the RGB-NIR cameras (tree canopy height, diameter, volume and NDVI) were compared with the ground measurements (tree canopy height, diameter, volume and LAI) by linear regression analysis using Costat, CoHort Software, Monterey, CA, USA). The residuals were calculated as the difference between individual tree parameters measured in the field and estimated from the UAV imagery. The root mean square error (RSME) of residuals was calculated along with the regression fit and the squared correlation coefficient between measured and estimated tree parameters. Bilinear regression analysis was used to determine the association between tree water status and the monthly canopy volume increment (SigmaPlot, Systat Software, Inc., San Jose, CA, USA).

## Results

**Fig 4:**
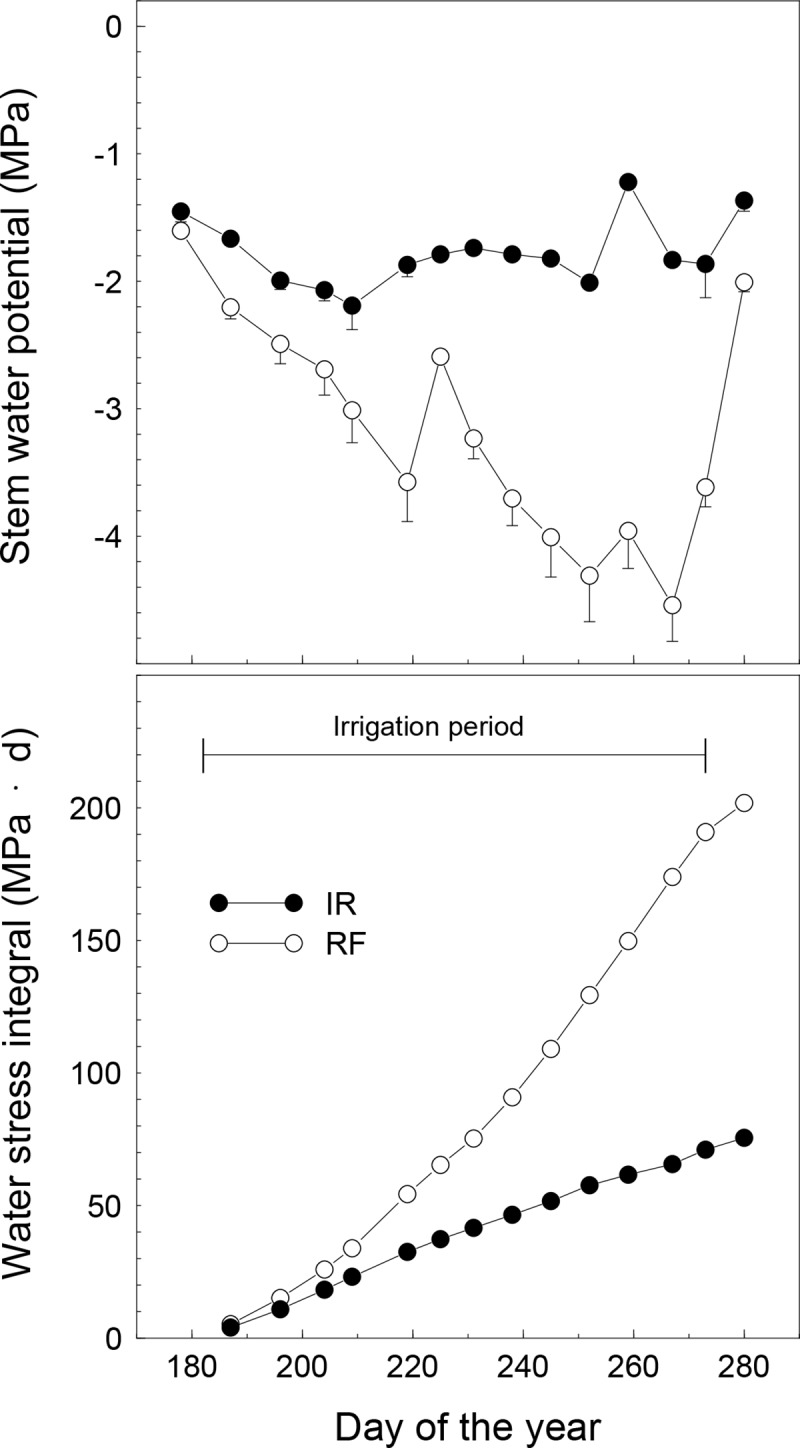
Seasonal course of stem water potential and water stress integral of irrigated (IR) or rainfed (RF) olive trees. Symbols are means of 9 (IR) or 6 (RF) trees. Vertical bars represent standard error calculated within each date of measurement. The horizontal line indicates the irrigation period.

The SWP of the irrigated trees was maintained above –2.2 MPa with an average of –1.8 MPa during the entire irrigation period in 2015, whereas the SWP of RF trees decreased progressively with increasing seasonal water stress and reached a minimum value of -4.5 MPa on DOY 267 (Fig 4A). The SWP of RF trees increased during the irrigation period (DOY 225) following a rainfall event, and then again on DOY 280 after the only irrigation applied to the RF trees (DOY 273) and three consecutive rainy days (DOY 275–277, a total of 45 mm). The degree of water deficit experienced by IR and RF trees, expressed as the water stress integral, was 73 and 190 MPa ˑd, respectively, at the end of the irrigation period (Fig 4B).

**Fig 5:**
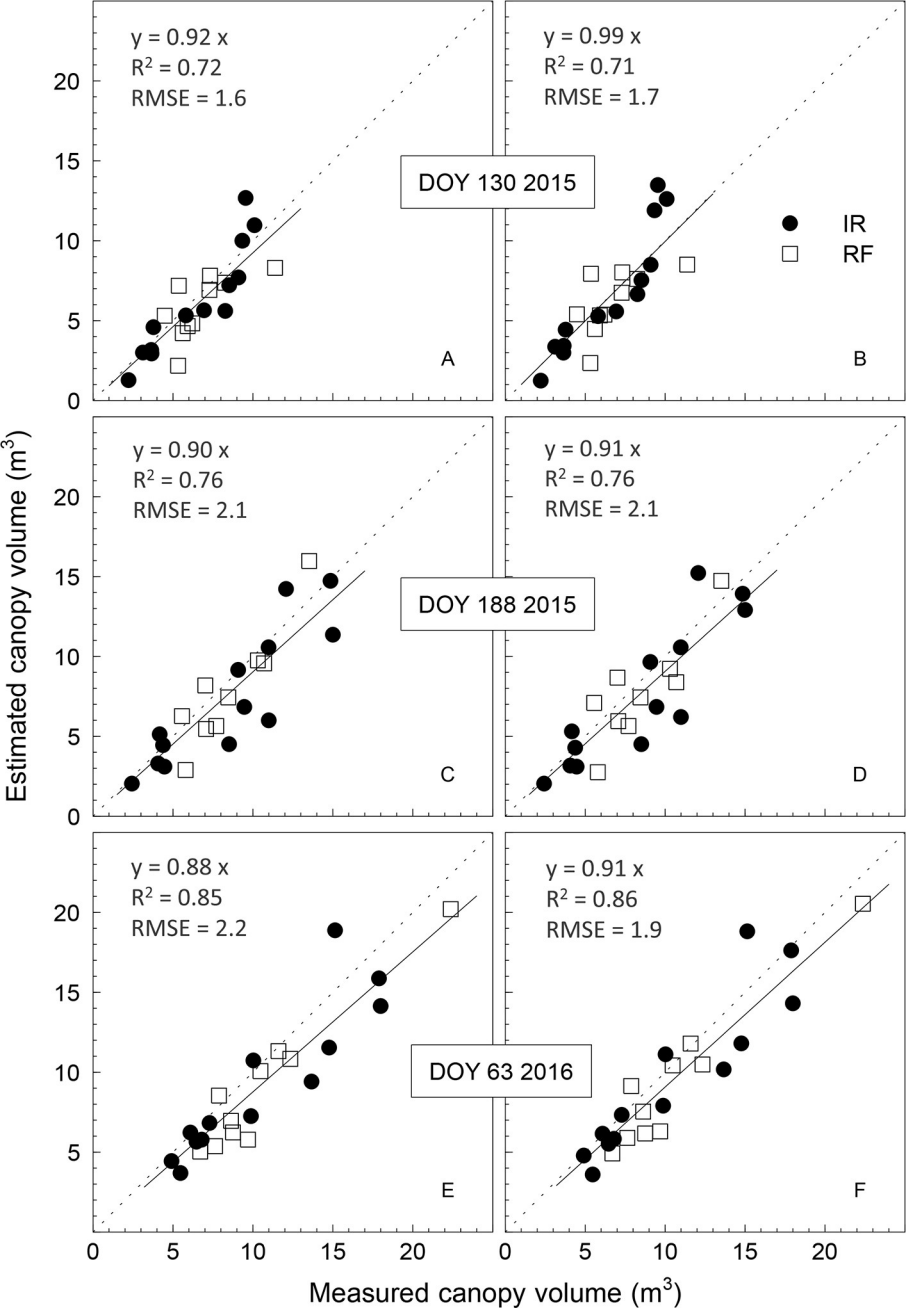
**Comparison between ground measured and UAV-estimated canopy volume of irrigated (IR) or rainfed (RF) olive trees** on DOY 130 (A, B) and 188 (C, D) in 2015 and DOY 63 in 2016 (E, F). The UAV-estimated canopy volumes were obtained by considering a distance of the canopy from the ground of 0.7 m (mean value for all trees during the study period) (A, C, E) or by using the distance measured for each tree (B, D, F). The flight altitude was either 70 m (DOY 130 and 188 in 2015) or 50 m (DOY 63 in 2016). No ground control points were used in the flight of DOY 130 in 2015, whereas 20 GCP were positioned in the orchard before the flights of DOY 188 in 2015 and DOY 63 in 2016. Each symbol represents one tree. The solid line is the fitted linear function forced through the origin and the dotted one is the 1:1 line.

The relationship between measured and estimated canopy volume on DOY 130 and 188 in 2015 and DOY 63 in 2016 was linear regardless of the different flight heights (50 and 70 m AGL) and processing procedures (with or without GCP). The canopy grew during the season, as it was evident by the increasing canopy volumes at the three dates of measurements. Canopy volume ranged between 2.2 and 11.4, 2.4 and 15.0, 4.9 and 22.4 m^3^, on DOY 130 and 188 in 2015 and DOY 63 in 2016, respectively (Fig 5A, 5C and 5E). The ability to estimate the canopy volume by the UAV-RGB camera technique was not affected by the use of GCPs (DOY 188 in 2015 and DOY 63 2016) as compared with the performance when they were not used (DOY 130 in 2015). Similarly, using the height of the canopy from the ground as a fixed average (d = 0.7 m) or as the actual height measured for each tree did not clearly affect the relationship between measured and estimated canopy volume. Only slight differences in R^2^ and RMSE values were observed when the two methods were compared (Fig 5A, 5B, 5E and 5F). Images acquired at the lowest flight altitude (50 m AGL) allowed to obtain the highest R^2^ value (0.86) and a low RMSE (1.9 m^3^) when the GCP and the measured distance of the canopy from soil were used for the 3D canopy reconstruction. In general, the estimated canopy volumes were slightly underestimated with respect to the ground measurements carried out on DOY 188 in 2015 and DOY 63 in 2016 (slope of the linear regression between 0.88 and 0.91), whereas an almost 1:1 ratio was observed on DOY 130 in 2015 (Fig 5B). Good correlations were also obtained between estimates and measurements of canopy height and diameter (Table 2). Tree height measured in the field on a total of 23 trees ranged between 2.10 m and 3.35 m, 2.18 m and 3.60 m, and 2.70 m and 3.90 m, on DOY 130 and 188 in 2015 and DOY 63 in 2016, respectively. The highest level of accuracy (RMSE = 0.16 m) in tree canopy height estimation was obtained on DOY 63 in 2016 (flight altitude of 50 m a.s.l. with GCP) with an R^2^ value of 0.87 and an almost 1:1 ratio of measured *vs* estimated canopy height. Low RMSE values were also found for the other two dates of flight (0.23 and 0.26 m on DOY 130 and 188 in 2015, respectively). By comparing the on-ground measured and the UAV-estimated canopy diameter, the coefficient of determination was 0.80, 0.81 and 0.84 for the UAV-images acquired on DOY 130 and 188 in 2015 and DOY 63 in 2016, respectively (Table 2). The highest and lowest RMSE values were observed on DOY 130 in 2015 and DOY 63 in 2016, respectively.

**Table 2:** Comparison between ground measured and UAV-estimated canopy height and diameter.

DOY	Year	Measured *vs* estimated tree canopy height (m)				Measured *vs* estimated tree diameter (m)			
		intercept	slope	R^2^	RMSE	intercept	slope	R^2^	RMSE
130	2015	0.34	0.92	0.73	0.23	-0.22	1.07	0.84	0.19
188	2015	0.57	0.77	0.73	0.26	-0.05	0.97	0.80	0.24
63	2016	-0.10	1.06	0.87	0.16	0.14	0.83	0.81	0.37

Canopy height and canopy diameter of olive trees were measured on DOY 130 and 188 in 2015, and DOY 63 in 2016. The flight altitudes were 70 m (DOY 130 and 188 in 2015) and 50 m (DOY 63 in 2016).

**Fig 6:**
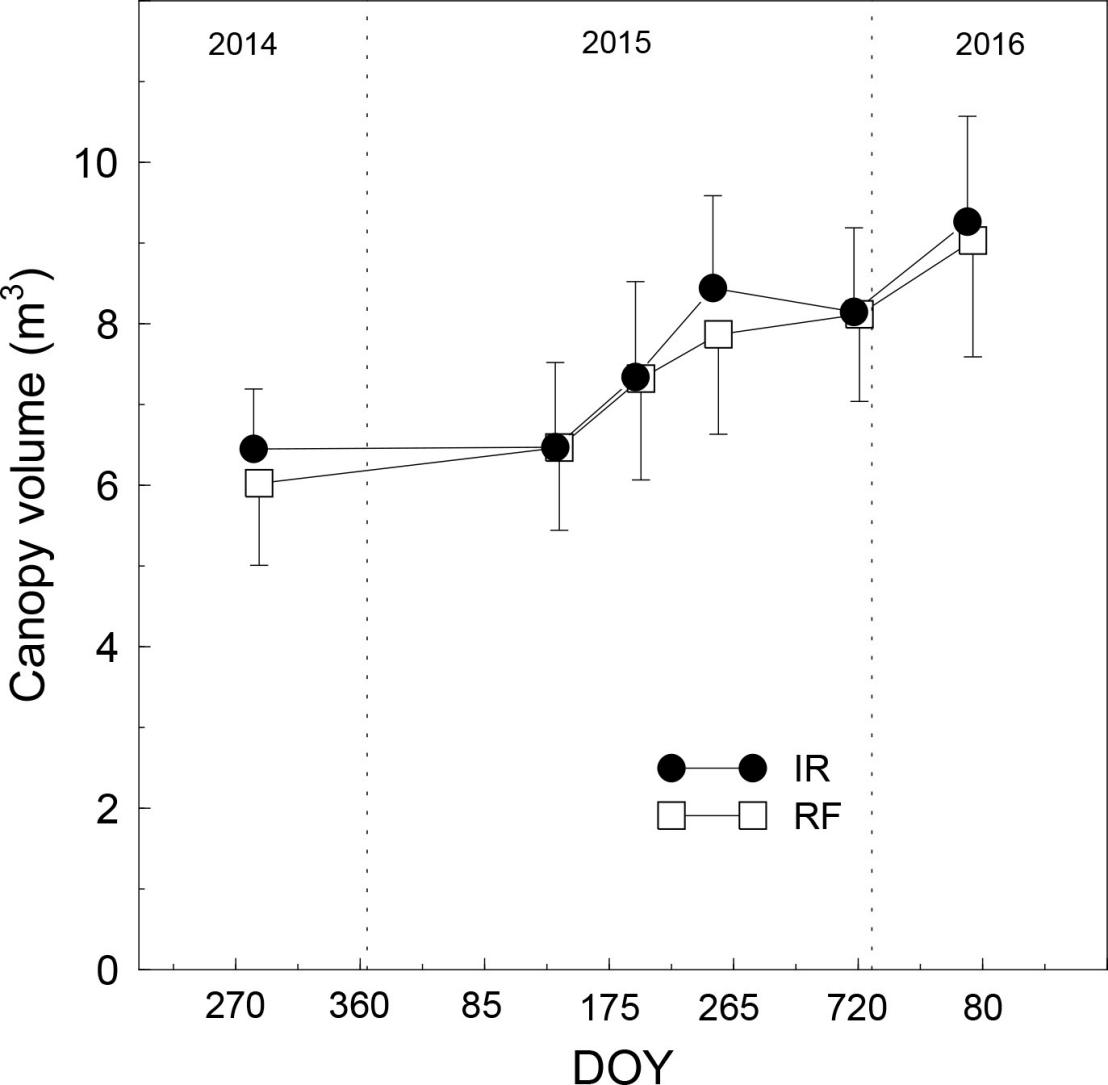
Canopy volume of irrigated (IR) or rainfed (RF) olive trees over three years. Symbols are means of 9 (FI) or 6 (RF) trees. Vertical bars represent standard error calculated within each date of flight.

Overall the seasonal courses of canopy growth monitored by UAV during the study period were consistent for both treatments, with the exception of two dates when mean canopy volume of the IR trees was slightly lower than the previous date (Fig 6). Differences in canopy volume or growth caused by the different soil water availability could also be detected, although only partially. The standard errors were always high due to the wide variability in tree size within each group. Thus, differences of canopy volume between irrigation treatments were, as expected, never significant.

**Fig 7:**
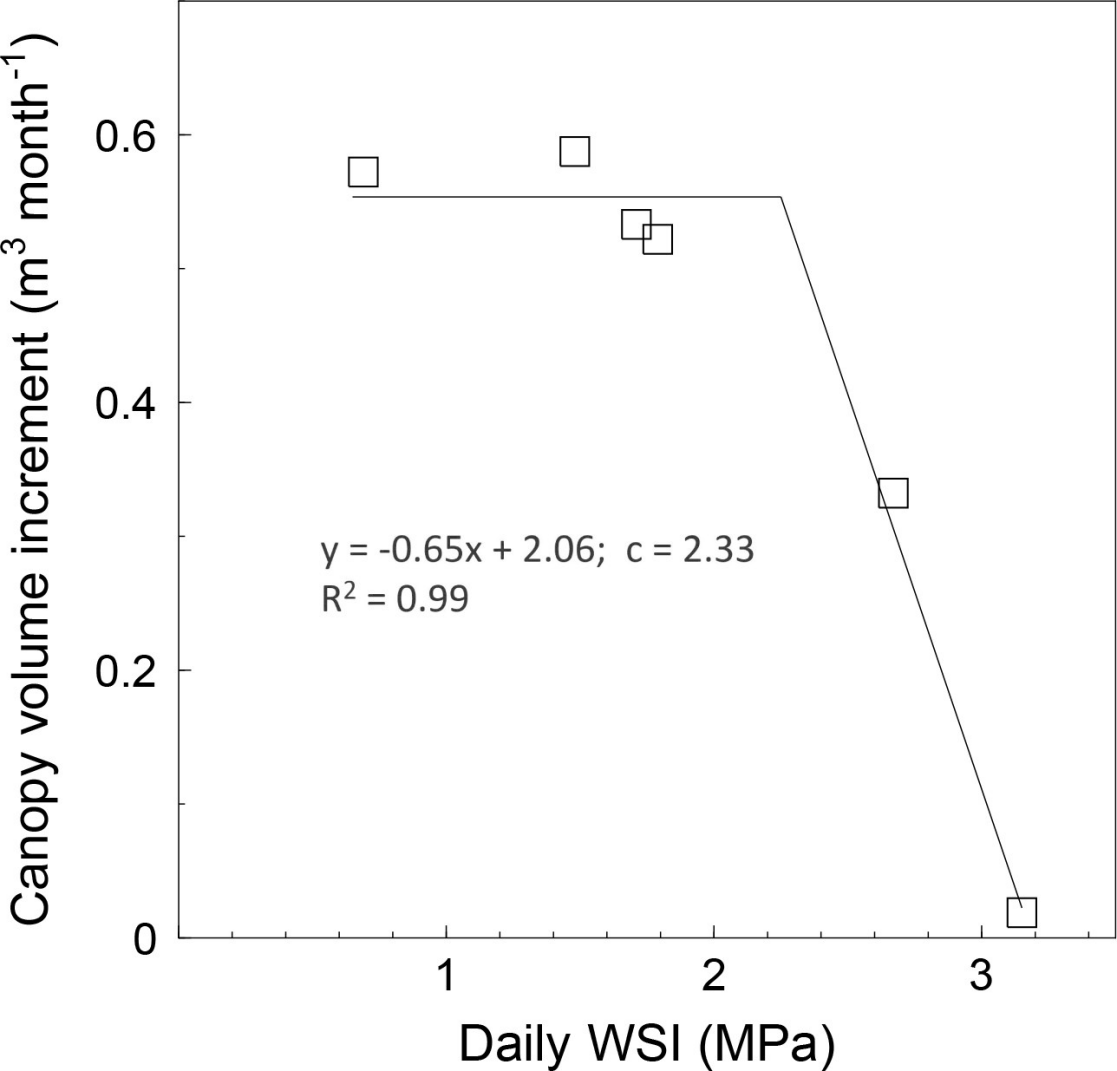
The relationship between tree water status and the monthly canopy volume increment of olive trees subjected to rainfed (RF) conditions. Tree water status was expressed as daily water stress integral (WSI) and the monthly canopy volume increment was estimated from DOY 130 through DOY 244 in 2015. Each symbol represents one tree. The parameter c is the breakpoint.

The monthly canopy volume increment between DOY 130 and 244 in 2015, estimated from UAV surveys, was highly correlated (R^2^ = 0.99) with the daily WSI of RF trees (Fig 7). On the contrary, the canopy volume increment of IR trees was not correlated with the daily WSI (data not shown).

**Fig 8:**
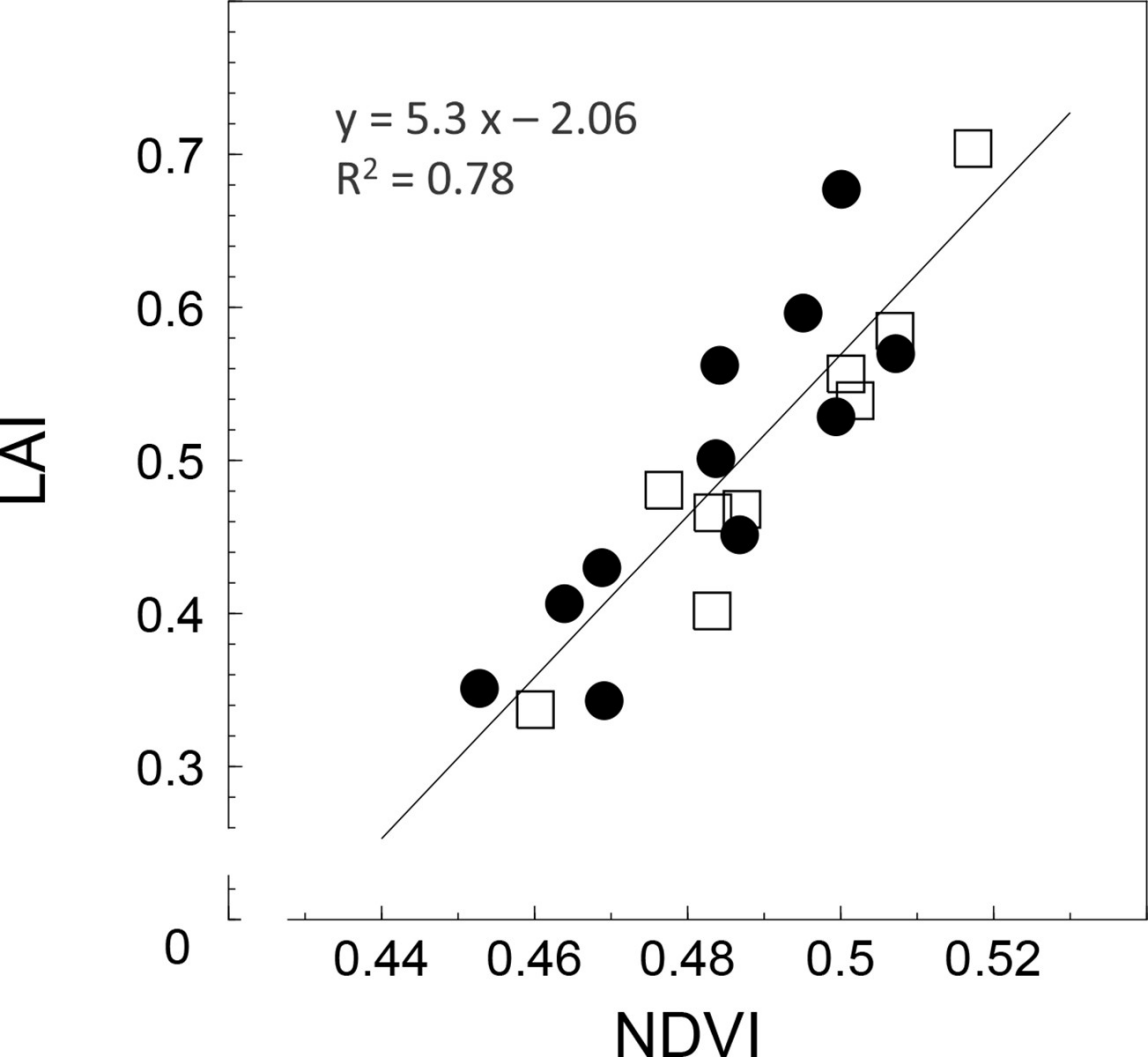
The relationship between NDVI and LAI in olive trees measured on DOY 130 in 2015 before the beginning of irrigation. The irrigation period lasted from DOY 182 through DOY 273 in 2015. Each symbol represents one tree. Different symbols are used to distinguish the two groups of trees before the beginning of the irrigation treatments. Black dots and white squares represent irrigated and rainfed trees, respectively.

The NDVI estimated on DOY 130 in 2015 (before the beginning of the irrigation period) was linearly correlated with both LAI and leaf chlorophyll measured on the same date (R^2^ = 0.78 and 0.80 for LAI and leaf chlorophyll, respectively) (Fig 8, S2 Fig). Values of NDVI and LAI in IR and RF trees, were comprised between 0.42 and 0.52, and 0.30–0.70, respectively.

**Fig 9:**
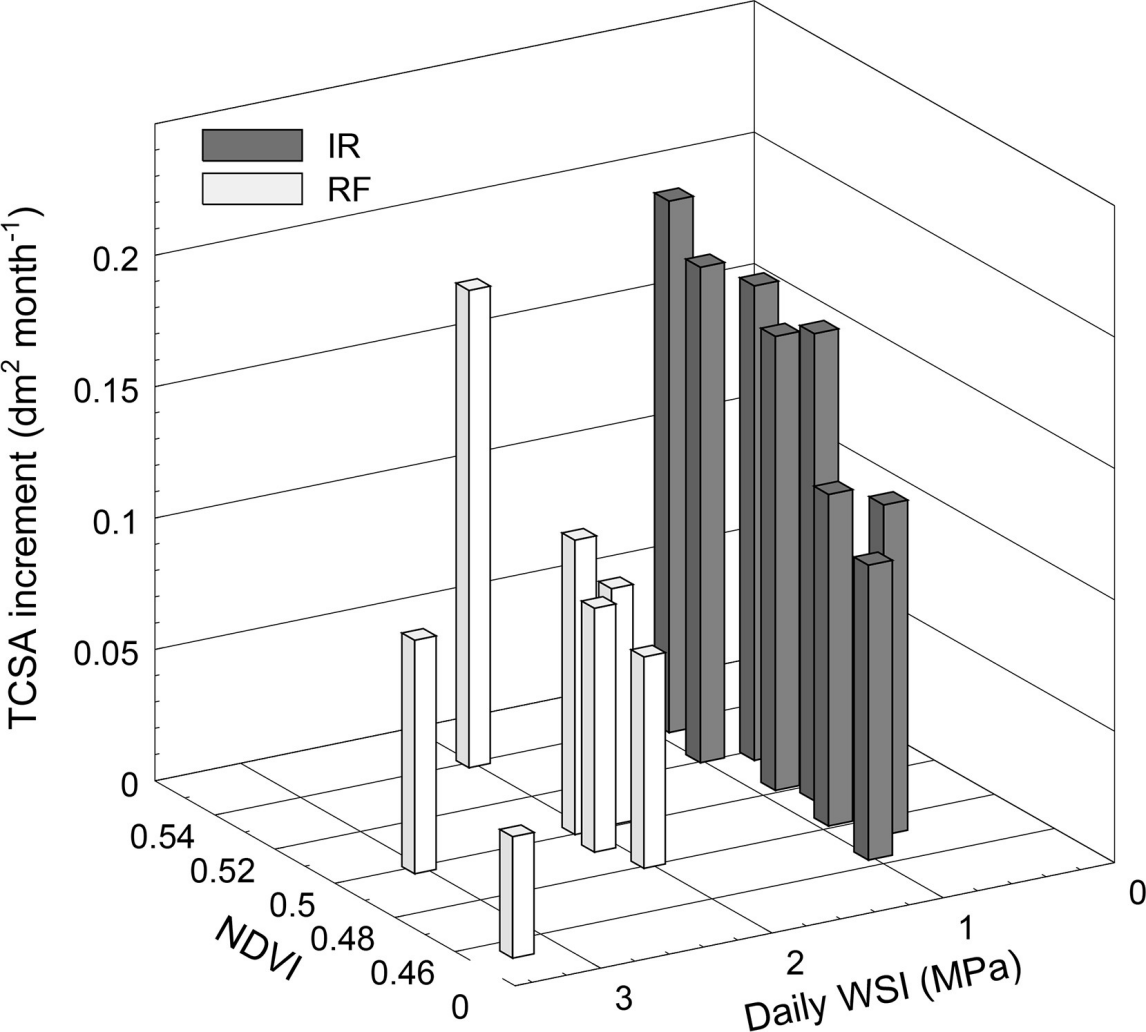
Trunk cross sectional area (TCSA) increment of olive trees with different water status and NDVI. Tree water status was expressed as daily water stress integral (WSI) during the irrigation season. Each bar represents one tree. The TCSA increment was calculated as the difference of TCSA measured on DOY 39 and DOY 314 in 2015. Multiple linear regression equation: Monthly TCSA increment = -0.03 daily WSI + 1.21 NDVI– 0.423; R^2^ = 0.87.

In both treatments, the monthly TCSA increment was affected by both the tree water status, expressed as daily WSI, and the tree vigour at the beginning of the year (DOY 39 in 2015) expressed as NDVI (Fig 9). Well irrigated trees showed a narrow range of water status (between -0.70 and -1.0 MPa), whereas the daily WSI of RF trees varied between -1.8 and -3.2 MPa. The highest monthly TCSA increments between DOY 39 and 314 in 2015 were measured in the most vigorous irrigated trees, whereas the lowest ones were observed on the less vigorous (low NDVI values) and more stressed (highest daily WSI value) RF trees. The combined effect of tree water status and vigour on TCSA increment was confirmed by the high value of the coefficient of determination (R^2^ = 0.87) of the multiple linear regression (Monthly TCSA increment = 0.03 daily WSI + 1.21 NDVI– 0.423).

## Discussion

The methodology proposed in this study is similar, in its first steps, to that used in previous experiments for the generation of the bare terrain surface (DTM) and the subsequent estimation of the canopy geometrical characteristics [37, 38]. Regardless of the height of flight, the number of GCPs used for the DSM generation and the height of the canopy from the ground (individual for each tree or mean orchard value) used for the calculation, the canopy volume was satisfactorily estimated. The correlations between the UAV-estimated and the on-ground-measured canopy volume of the individual trees showed coefficient of determination and RMSE values always comprised between 0.71 and 0.86 and 1.6 m^3^ and 2.1 m^3^, respectively. A previous study conducted on mature olive trees in Spain reported lower coefficient of determination between estimated and measured canopy volumes by using images taken at an altitude of 50 m (R^2^ = 0.65) and 100 m (R^2^ = 0.63) [27]. In our study, R^2^ increased substantially (from 0.76 to 0.85) by reducing the flight altitude from 70 to 50 m. The slight underestimation observed in the UAV-estimated canopy volume can be reasonably interpreted as a higher accuracy with respect to the on-ground-measured values. In fact, while the SfM technique can detect (and exclude) the empty volume in the central part of the olive tree canopy, typical of the vase training system, the ellipsoid method used for the on ground canopy measurement cannot. Previous studies on the canopy geometry of olive and eucalypt trees confirm this hypothesis [16, 38]. In both studies, by comparing the canopy volumes estimated through the ellipsoid method with those estimated by laser scanner technique (LIDAR), which actually represents the most precise technology available for this purpose, an overestimation of the ellipsoid method was observed [16, 39]. A possible limitation of the methodology presented here is that it requires the value of the mean height of the canopy from the ground as input data for canopy volume estimation. This information is easier to determine in modern, intensive olive orchards than in traditional, irregular olive orchards in which olive trees are irregular in age and/or training system.

The estimation of specific geometric parameters by a fully-automated procedure also proved successful. Regardless of flight altitude, both tree height and crown diameter showed RMSE values comprised between 5 and 9% and between 8 and 13%, respectively. These results are comparable to those obtained in other studies where both the SfM technique and airborne laser scanner technology were used to estimate those parameters [27, 40, 41]. In particular, by using a consumer camera modified for colour infrared detection, mounted on a fixed-wing UAV, it was possible to estimate the tree canopy height and diameter of isolated mature olive trees in Spain with an accuracy of about 12% [28] and 19% [27], respectively.

Although only two (DOY 188 in 2015 and DOY 63 in 2016) of the six flights performed had been specifically scheduled for the DSM generation (presence of GCPs on the ground), the canopy volume was calculated for all flights in order to draw the seasonal patterns of canopy growth for both irrigated and rainfed trees (Fig 6). The seasonal courses were quite representative of the natural canopy growth and the effect of water stress on vegetative growth seemed evident in correspondence of the maximum level of stress experienced by the rainfed trees. There were no significant differences between the two treatments due to the high variability in vigour within each treatment. The effect of water deficit was more evident when the monthly canopy volume increment of each rainfed tree was plotted against the water stress experienced by the same tree (daily WSI) (Fig 7). A reduction in canopy volume induced by water stress has been observed in previous studies conducted on different olive cultivars, including Frantoio [1, 6, 10, 42].

One of the advantages of using miniaturized cameras is that two or more devices can be transported contemporarily by the UAV, allowing the acquisition of different images types. In this study, the multispectral images acquired in February and May 2015 were used to calculate NDVI at the single tree level for both dates of flight. A clear correlation (R^2^ = 0.78) between NDVI and LAI was observed in May, confirming previous findings on olive trees [24, 25]. The combined effect of the NDVI (vigour) and the daily WSI (water status) on the TCSA increment was evident. When both factors were considered separately the coefficient of determination with the TCSA increment were 0.57 and 0.55 for NDVI and ISWP, respectively. However, when they were combined in a multilinear regression the R^2^ increased significantly (R^2^ = 0.87). This result suggests that the individual tree vigour should be always accounted for in field trials aimed at evaluating the single effect of agronomical practices on tree vegetative parameters.

The information derived from NIR-visible images can be used to improve irrigation management, fruit and oil quality and to estimate biomass production and carbon sequestration both at tree and field level. The use of more representative values of canopy diameter obtained from RGB images also allows a better estimate of K_r_ [29], whereas vegetation indices form multispectral images can be used to derive specific K_c_ values from olive orchard under different conditions [43]. Better estimates of crop water requirement can be achieved by using these improved methods to estimate K_r_ and K_c_. Moreover, the possibility of estimating the tree canopy volume throughout the growing season allows to adopt pruning strategies that improve the light interception at field level and light distribution within the crown. Recent studies reported that fruit and oil quality is directly affected by the amount of light intercepted by the fruits which, in turn, depends on the shape and size of the canopy [44, 45]. Finally, the high level of accuracy and time saving in estimation of tree canopy volume using the methodology described in this work, make UAV, RGB images and SfM techniques feasible for the above-ground biomass determination and, through specific correlation, the carbon sequestration at tree and field scale [46, 47].

## Conclusions

In this study we showed that UAV, RGB-NIR cameras and SfM techniques can be used to effectively estimate biophysical and geometrical parameters such as LAI, tree height, canopy diameter and canopy volume of olive trees. The method we propose for the tree canopy volume estimation, based on the processing of DTM and DSM raster files, has been previously used for the canopy height identification. The UAV imagery was successfully used to assess the effects of different soil water availability on canopy growth. These results show promising perspective in the use of this technique in field experiments on irrigation management. Further investigations are required to verify whether the good results of accuracies assessment obtained in a flat olive orchard can also be confirmed on slopes.

## Supporting information

S1 Fig The relationship between SPAD and leaf chlorophyll. Data were acquired on DOY 130 in 2015 in olive trees before the beginning of the irrigation treatments (irrigation *vs* rainfed). Each symbol represents one tree.
(PDF)

S2 Fig The relationship between NDVI and leaf chlorophyll on DOY 130 in 2015. Irrigation period lasted from DOY 182 through DOY 273 in 2015. Each symbol represents one tree. Different symbols are used to distinguish the two groups of trees before the beginning of the irrigation treatments. Filled dots and open symbols represent irrigated and rainfed trees, respectively.
(PDF)
