# Supplementary figures and images for: High-resolution imagery acquired from an unmanned platform to estimate biophysical and geometrical parameters of olive trees under different irrigation regimes
Source: PLoS One. 2019 Jan 22;14(1):e0210804. doi: 10.1371/journal.pone.0210804 (PMC6342295; doi:10.1371/journal.pone.0210804)

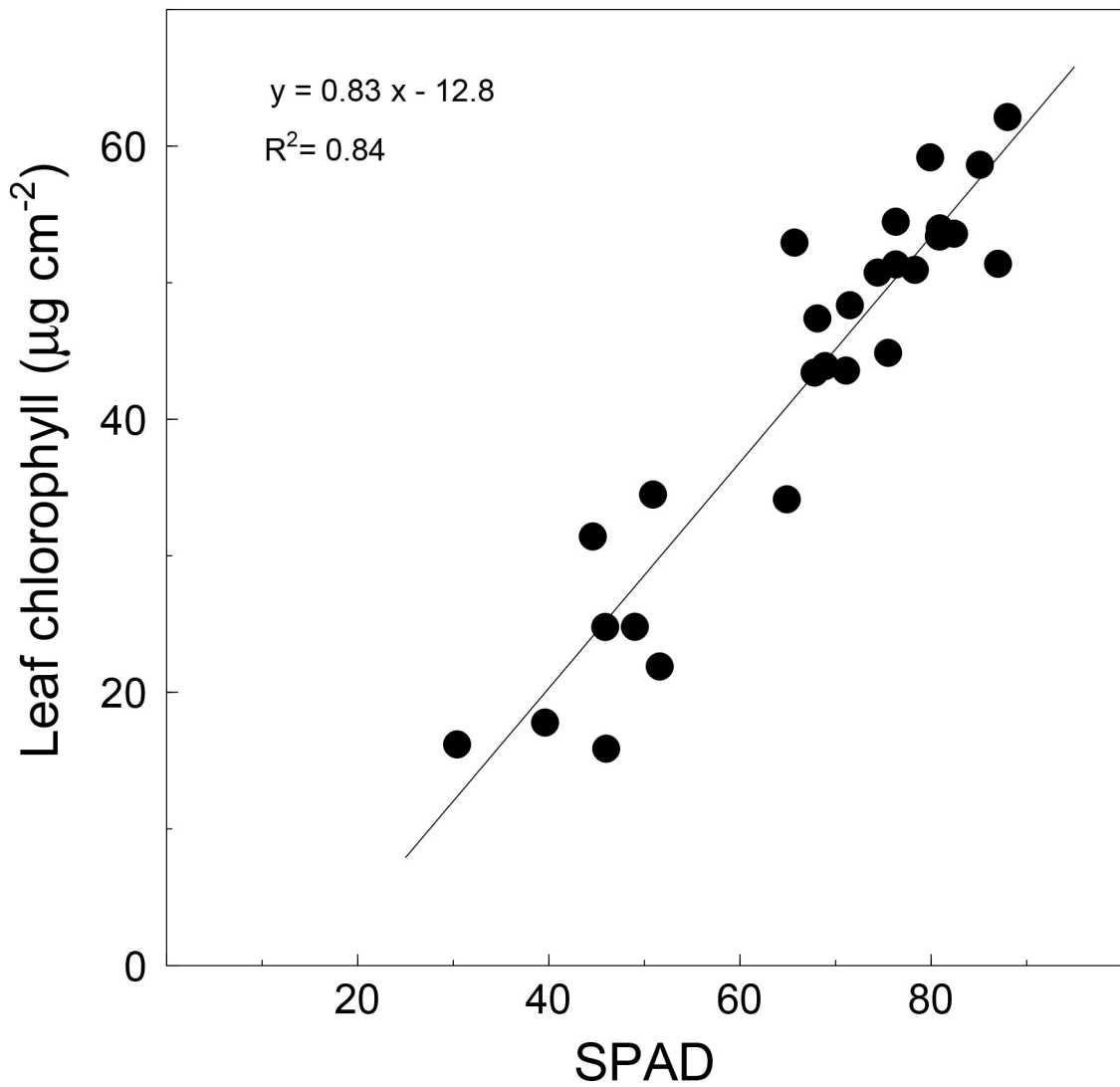

Supplement: S1 Fig — Data were acquired on DOY 130 in 2015 in olive trees before the beginning of the irrigation treatments (irrigation vs rainfed). Each symbol represents one tree. (PDF) [file pone.0210804.s001.pdf]

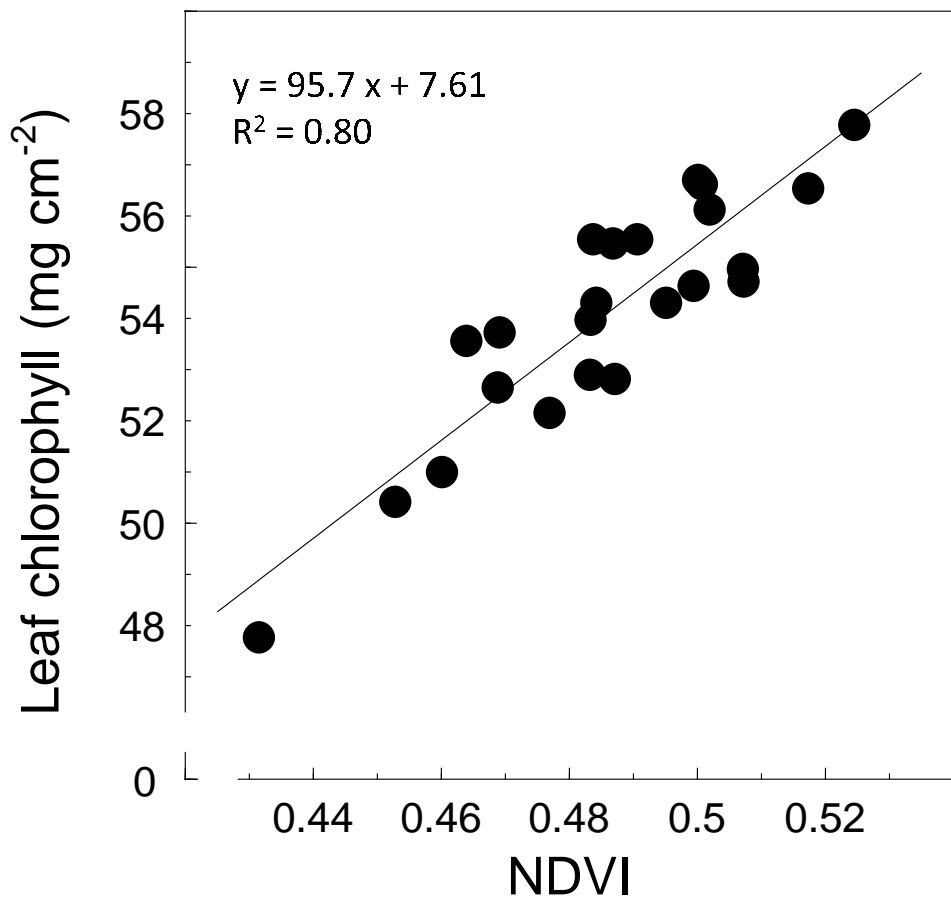

Supplement: S2 Fig — Irrigation period lasted from DOY 182 through DOY 273 in 2015. Each symbol represents one tree. Different symbols are used to distinguish the two groups of trees before the beginning of the irrigation treatments. Filled dots and open symbols represent irrigated and rainfed trees, respectively. (PDF) [file pone.0210804.s002.pdf]
